# Differences in itch and pain behaviors accompanying the irritant and allergic contact dermatitis produced by a contact allergen in mice

**DOI:** 10.1097/PR9.0000000000000781

**Published:** 2019-09-11

**Authors:** Zhe Zhang, Nathalie M. Malewicz, Xiaoyun Xu, Jianhao Pan, Nina Kumowski, Tao Zhu, Steven G. Shimada, Hong Nie, Robert H. LaMotte

**Affiliations:** a Department of Anesthesiology, Yale University School of Medicine, New Haven, CT, USA; b Guangdong Province Key Laboratory of Pharmacodynamic Constituents of TCM and New Drugs Research, College of Pharmacy, Jinan University, Guangzhou, China; c Department of Anesthesiology and Intensive Care Medicine, University Duisburg-Essen, University Hospital Essen, Essen, Germany

**Keywords:** Itch, Pain, Hyperalgesia, Allodynia, Inflammation, ICD, ACD, Dermatitis

## Abstract

**Introduction:**

Irritant contact dermatitis (ICD) and allergic contact dermatitis (ACD) are inflammatory skin diseases accompanied by itch and pain. Irritant contact dermatitis is caused by chemical irritants eliciting an innate immune response, whereas ACD is induced by haptens additionally activating an adaptive immune response: After initial exposure (sensitization) to the hapten, a subsequent challenge can lead to a delayed-type hypersensitivity reaction. But, the sensory and inflammatory effects of sensitization (ICD) vs challenge of ACD are insufficiently studied. Therefore, we compared itch- and pain-like behaviors and inflammatory reactions evoked in mice during the sensitization (ICD) vs challenge phase (ACD) of application of the hapten, squaric acid dibutylester (SADBE).

**Objectives:**

Our aim was to compare itch- and pain-like behaviors and inflammatory reactions evoked in mice during the sensitization (ICD) vs challenge phase (ACD) of application of the hapten, squaric acid dibutylester (SADBE).

**Methods:**

Mice were sensitized on the abdomen with 1% SADBE (ACD) or vehicle treated (ICD, control). Spontaneous and stimulus-evoked itch- and pain-like behaviors were recorded in mice before and after 3 daily challenges of the cheek with 1% SADBE (ACD, ICD). Cutaneous inflammation was evaluated with clinical scoring, ultrasound imaging, skin thickness, histology, and analyses of selected biomarkers for contact dermatitis, IL-1β, TNF-α, CXCL10, and CXCR3.

**Results:**

Allergic contact dermatitis vs ICD mice exhibited more spontaneous site-directed scratching (itch) and wiping (pain). Allergic contact dermatitis—but not ICD—mice exhibited allodynia and hyperalgesia to mechanical and heat stimuli. Inflammatory mediators IL-1β and TNF-α were upregulated in both groups as well as the chemokine receptor, CXCR3. CXCL10, a CXCR3 ligand, was upregulated only for ACD. Inflammatory responses were more pronounced in ACD than ICD.

**Conclusion:**

These findings provide new information for differentiating the behavioral and inflammatory reactions to hapten-induced ICD and ACD.

## Abstract 2

Supplemental Digital Content is Available in the Text.

## 1. Introduction

Irritant contact dermatitis (ICD) and allergic contact dermatitis (ACD) are inflammatory diseases of the skin accompanied by sensations of itch and pain that diminish the quality of life.^24,34,46^ Allergic contact dermatitis is a type IV delayed hypersensitivity reaction mediated by allergen specific T cells.^13,34^ On first skin contact, the hapten is incorporated by immature dendritic cells and transported to the lymph nodes to prime T cells (sensitization phase).^3,20^ Subsequent exposure to the same allergen (challenge or elicitation phase) triggers invasion of these T cells, inducing apoptosis of keratinocytes and resulting in ACD.^28,36^

Irritant contact dermatitis is mediated by direct damage to the keratinocytes by the intrinsic toxicity of an irritating substance. There is a rapid activation of the innate immune system causing the release of cytokines, including interleukin-1-beta (IL-1β), interleukin-6 (IL-6), tumor necrosis factor (TNF-α), and chemokines, resulting in a proinflammatory milieu in the skin.^8,9,25,26^ Irritant contact dermatitis can occur as a distinct phenomenon and separately definable from ACD. But, a proinflammatory milieu is essential for the sensitization and development of ACD.^24^ Indeed, the intensity of the inflammation of ACD is determined by the magnitude of the cutaneous irritation or “induced ICD” produced during the previous sensitization phase.^2^

In a murine model of ACD (called contact hypersensitivity) mice previously sensitized to squaric acid dibutylester (SADBE) exhibited itch- and pain-like behaviors directed towards a subsequently challenged area of ACD on the cheek or calf.^13,34^ Electrophysiological recordings and calcium imaging of cell bodies of pruriceptive nociceptive neurons innervating the area of ACD revealed an increase in membrane excitability, increased density of voltage-gated sodium current and de novo responses to C-X-C motif chemokine 10 (CXCL10), a chemokine expressed by keratinocytes that attracts antigen specific T cells to the area of hapten challenge.^11,34^ Proinflammatory cytokines, such as IL-1β and TNF-α, were hypothesized to act to enhance neuronal excitability and sensitivity to nociceptive stimuli. But, measurements of these cytokines in the skin and behavioral measures of hyperalgesia and allodynia accompanying ACD were not obtained during the sensitization and challenge phases of SADBE-induced ACD. In addition, no measurements of spontaneous itch- and pain-like behaviors were obtained during the sensitization phase.

Here, we investigated whether SADBE-induced ICD vs ACD inflammation and cytokine upregulation are accompanied by differences in behavioral signs of itch, pain, hyperalgesia, and allodynia.

## 2. Methods

Detailed descriptions of methods are to be found in the supplement (available as supplemental digital content at http://links.lww.com/PR9/A50).

### 2.1. Animals

C57BL/6 SPF mice (6–8 weeks old, male: 20–25 g, female 15–20 g, Charles River Laboratories) were used at Yale for all experimental tests on the cheek. Female mice were used in behavioral tests of spontaneous itch- and pain-like behaviors. All experimental procedures were approved by the Institutional Animal Care and Use Committee of Yale University.

Experiments on the calf (thickness, histology, and expression of protein and mRNA) were performed on C57BL/6 SPF male mice (weight: 20–25 g) at the Medical Science Experimental Animal Center of Guangdong Province in China. Experimental procedures were in accordance with the Guide for the Care and Use of Laboratory Animals at Jinan University. All experiments were in accordance with the National Institutes of Health (NIH) and the International Association for the Study of Pain (IASP) guidelines.

Mice were housed under a 12-hour light/dark cycle with free access to standard laboratory food and tap water. All experiments were performed and evaluated by experimenters blinded as to the experimental conditions.

### 2.2. Models of irritant and allergic contact dermatitis

**Figure 1.**
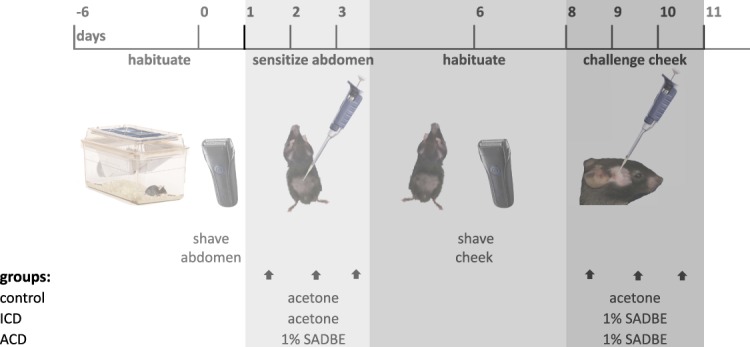
The experimental procedures for each group of control, ICD, and ACD mice tested on the cheek. The same procedure was applied in experiments using skin from the calf with the exception that the calf was challenged with acetone or 1% SADBE only on days 8 and 9. ACD, allergic contact dermatitis; ICD, irritant contact dermatitis; SADBE, squaric acid dibutylester.

Irritant contact dermatitis and ACD models were produced with the same protocol throughout all experiments (Fig. 1). Calf skin was used for the histology and measurements of mRNA and protein because it yielded more tissue than the cheek.

Before testing, mice were acclimatized for 7 days to the housing facility, followed by the daily habituation. Under brief anesthesia with isoflurane (2% in pure oxygen), the abdominal skin (2 × 1-cm area) and the right cheek or calf were shaved with mice positioned on a warming pad set to 37°C (Kent Scientific,Torrington, CT). Mice were sensitized by 25-μL topical 1% SADBE (Sigma, St. Louis, MO) in acetone onto the abdominal skin once daily for 3 consecutive days for the ACD group, whereas ICD and control groups were treated with 25 μL of acetone vehicle. Five days later, on day 8, the right cheek or calf of ICD and ACD mice was challenged for 2 or 3 consecutive days once daily with 25-μL topical 1% SADBE in acetone. The control group was treated with 25 μL of acetone vehicle.

### 2.3. Spontaneous behaviors directed toward the cheek

All behavioral tests were performed as described^21,40^ on the cheek before challenge and again 24 hours after each of 3 challenges (first, second, and third) (Fig. 1).

### 2.4. Responses to mechanical and heat stimulation of the cheek

The mechanical stimuli consisted of nylon filaments with tip diameters in µm (and delivering bending forces in mN) of 67 (0.23), 100 (2), 100 (10), and 100 (20). Heat stimuli were applied by means of a probe consisting of a chip resistor (2 × 3 mm) with a thermocouple (used to servocontrol temperature at the skin probe interface to within ±0.1°C)^42^ and preset for contact with skin to either 38°C for warmth or 52°C for noxious heat. Tests were performed before and 24 hours after first, second, and third SADBE challenge.

Behavioral reactions to stimuli were numerically ranked based on aversiveness according to published methodologies,^49,51^ no response = 0, detection (nonaversive) = 1, mildly aversive (withdrawal) = 2, and strongly aversive (escape/attack) = 3.^49^ In case of prolonged aversive behavior (wiping), the total number of stimulus-evoked, ipsilateral wipes of the check with the forelimb was added to the score. The sum of scores after 10 stimulations per stimulus (“total response”) was calculated per mouse.^51^ Allodynia was defined as an increased total response to the innocuous stimuli (38°C heat and 0.23-mN filament), whereas hyperalgesia was defined as increased total response to the nociceptive stimuli of 52°C heat or the 10- and 20-mN filaments.

### 2.5. Scoring the severity of inflammation by measuring erythema, scaling, and skin-fold thickness

Photographs of the cheek of each mouse were obtained under standardized lighting conditions. The severity of the inflammation based on daily photographs of the cheek was rated before and after challenge for the amount of erythema and scaling (desquamation), each assessed independently: 0, none; 1, slight; 2, moderate; 3, marked; and 4, very marked.^12,48^ Than under brief anesthesia, the skin-fold thickness was measured 3 times using a micrometer (Mitutoyo, Tokyo, Japan), and the mean calculated.

### 2.6. Ultrasound images of cheek skin, in vivo

A high-frequency ultrasound scanner (VisualSonics Vevo 770) in B mode (17-Hz frame rate, 100% power) with a 55-MHz transducer (RMV 708) was used for imaging the superficial skin with optimal spatial resolution. To evaluate changes in blood flow, such as vasodilation, the power Doppler mode was used. Hereafter, in the ultrasound images, the total colored area, signaling blood flow, under exclusion of prominent vessels (>0.5 mm), was measured with ImageJ (NIH, Bethesda, MD) with a color deconvolution plug-in.^37,39^ The areas were analyzed using an ImageJ, macromeasuring regions of interest, in a designated area of the images.^29^

Ultrasound images with distinguishable skin layers were also analyzed for the overall skin thickness of different layers including the stratum corneum, epidermis, dermis, and hypodermis using ImageJ. Skin layers were distinguished based on their order, appearance, and echogenicity with light structures characterized as hyperechoic, white, or hypoechoic, gray, or darker areas.^15^

### 2.7. Hematoxylin and eosin staining

Mice were killed 24 hours after the second challenge, and the treated calf skin (1 × 1 cm) was harvested and fixed in 4% formaldehyde.

Hematoxylin stained areas were analyzed using ImageJ and number of cells quantified.^29,37,39^

### 2.8. Immunohistochemistry

Immunohistochemistry was performed by Shanghai Gefan Biotechnology.co, Ltd, using the company's standard protocol.^43,44^ All images were captured with a digital camera DM6000 (Leica, Wetzlar,Germany). For quantitative analysis, the positive area (brown area) vs the lilac area of the images was measured with ImageJ^37,39^ and the percentage of positively stained area per tissue calculated.^29^

### 2.9. Real-time quantitative polymerase chain reaction

The mRNA levels of TNF-α, IL-1β, CXCL10, and CXCR3 in calf skin were measured through real-time quantitative polymerase chain reaction (RT-qPCR). Mice were killed 24 hours after the second challenge, and the treated calf skin of the mice was collected for later mRNA analysis. The primers used are listed in Table 1. The RT-qPCR conditions were 95°C for 30 seconds, followed by 40 cycles of 95°C for 5 seconds and 60°C for 30 seconds. The mRNA levels of all target genes were normalized with those of β-actin and quantified using the 2^−ΔΔCT^ method.^38^

**Table 1:**
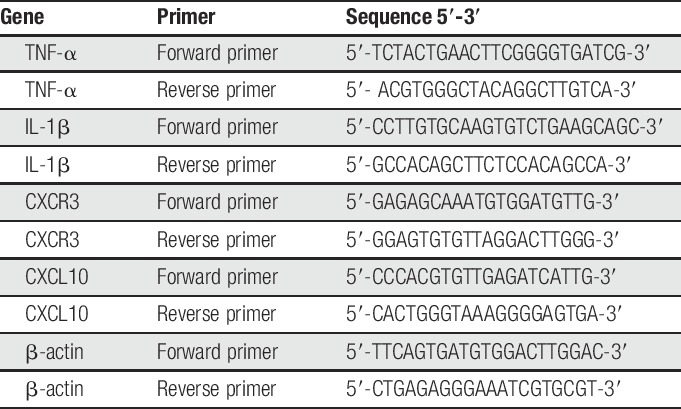
Primers used for real-time quantitative polymerase chain reaction (PCR).

### 2.10. Statistical analysis

Differences between mean values were analyzed with a mixed-design analysis of variance (ANOVA), in case of repeated days of testing in the same animals with repeated-measures ANOVA (RMANOVA). The histological analysis and the expressions of mRNA and protein levels were analyzed through 1-way ANOVAs.

Differences between mean of number of bouts of scratching or number of wipes were analyzed with a mixed-design RMANOVA, with repeated measures over days of testing. Differences in the mean total response derived from responses of male mice to each von Frey filament and each heat stimulus were separately analyzed with a two-way RMANOVA with repeated measures over 4 days of testing. The effects of force and temperature on responses to the mechanical and heat stimuli, respectively, were separately analyzed with a mixed-design RMANOVA. The erythema score, scaling score, skin-fold thickness, and skin thickness of different skin layers and power Doppler evaluation of ultrasound images were analyzed with a two-way ANOVA. All F-values and *P*-values for interaction are shown in Supplemental Table 1 (available as supplemental digital content at http://links.lww.com/PR9/A50). Each of the ANOVAs was followed by Bonferroni corrections for testing between individual means. A value of *P* < 0.05 was considered as statistically significant. Figures are shown as mean ± SEM and SD for categorical variables.

## 3. Results

### 3.1. Spontaneous itch- and pain-like behaviors

**Figure 2.**
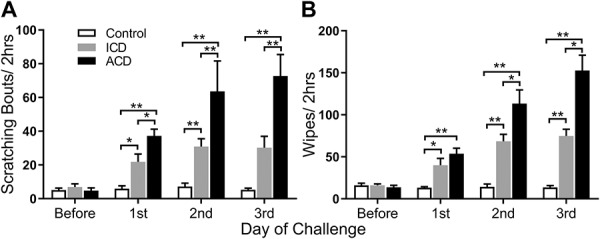
Effects of ICD and ACD in eliciting spontaneous scratching and wiping behaviors in male mice. Mean bouts of spontaneous scratching (A) and mean number of wipes (B) were obtained before and 24 hours after the first, second, and third challenge with SADBE in mice that were previously sensitized to the chemical (ACD) or previously exposed only to the acetone vehicle (ICD). Control mice received only the acetone before and during challenge. *P < 0.05, **P < 0.01, error bars: SEM. n = 12 mice/group. ACD, allergic contact dermatitis; ICD, irritant contact dermatitis.

Male mice with either ACD or ICD exhibited a significantly greater mean number of spontaneous scratching bouts and wipes after each challenge with SADBE than they did before challenge (Fig. 2). Control mice treated with the acetone vehicle exhibited no change in spontaneous scratching or wiping after each day of testing. The amount of scratching and wiping in both ACD and ICD groups was significantly greater after the first, second, and third challenges than before challenge. Allergic contact dermatitis mice had significantly more scratching bouts than ICD mice after each challenge and also more wipes but only after the second and third challenges.

To investigate sex differences in these spontaneous behaviors, the same behavioral experiments were performed with females. The results obtained from female mice were virtually identical to those obtained from males with 1 exception: After ACD, females exhibited significantly more spontaneous wiping than males after the second and third challenges (supplemental Fig. 1, supplemental Table 1, available as supplemental digital content at http://links.lww.com/PR9/A50). The sex of the mice did not influence the differences between groups for both wiping and scratching behavior.

### 3.2. Behavioral responses to mechanical stimuli

**Figure 3.**
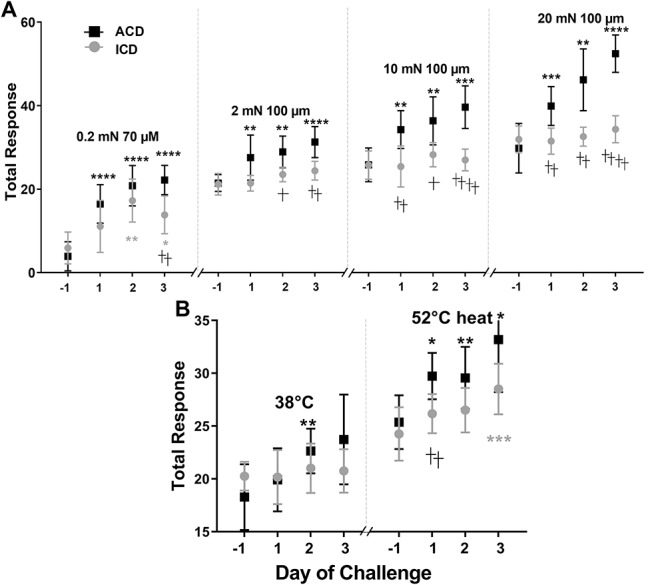
Effects of ACD and ICD on behavioral reaction (“total response”) to von Frey filaments and a heated contact thermode applied to the cheek. Each stimulus was applied before and 24 hours after each SADBE challenge on the cheek in ICD and ACD groups of male mice. (A) Mean total response to an innocuous von Frey filament of 0.23 mN and mean DS to aversive filaments of 100-µm tip diameter applied at 3 different bending forces (2, 10, and 20 mN). (B) Mean total response to contact with a thermode having an innocuous temperature of 38°C and a contact thermode having a noxious temperature of 52°C. *P < 0.05, **P < 0.01 , ***P < 0.001, ****P < 0.0001 for significant differences between before and after challenge within a group, whereas †*P* < 0.05, ††*P* < 0.01, †††P < 0.001, ††††P < 0.0001 indicate significant differences between the 2 groups. Error bars: SD. n = 12 male mice for ICD, n = 11 for ACD. ACD, allergic contact dermatitis; ICD, irritant contact dermatitis.

The mean total response significantly increased with bending force for both ACD and ICD groups both before and after challenge. In relation to scores obtained before challenge, the mean total response of ACD mice was consistently greater after each challenge with SADBE both in response to the innocuous filament of 0.23 mN (allodynia) and to each of the normally aversive filament forces of 2 to 20 mN (hyperalgesia) (Fig. 3A). By contrast, ICD mice exhibited significantly lesser allodynia but only after the second and third challenges and no hyperalgesia to filaments of 2 to 20 mN.

### 3.3. Behavioral responses to heat

For both ICD and ACD, the mean DS before and after treatment was significantly greater in response to 52°C than to 38°C. In response to the innocuous temperature of 38°C, ACD- and ICD-elicited similar mean total responses both before and after the first and third challenge with SADBE (Fig. 3B). But, after the second challenge, ACD but not ICD mice exhibited a significant increase in the response to this innocuous stimulus (allodynia).

In response to the noxious heat of 52°C, the mean total response before challenge was the same for both groups before SADBE challenge and significantly greater than that elicited by innocuous heat. The responses were significantly increased after each challenge for ACD mice, hyperalgesia but for the ICD mice only after the fourth treatment and to a significantly lesser magnitude. Thus, ACD produced a more immediate and significantly greater magnitude of hyperalgesia than ICD.

### 3.4. Clinical assessment of skin reactions

**Figure 4.**
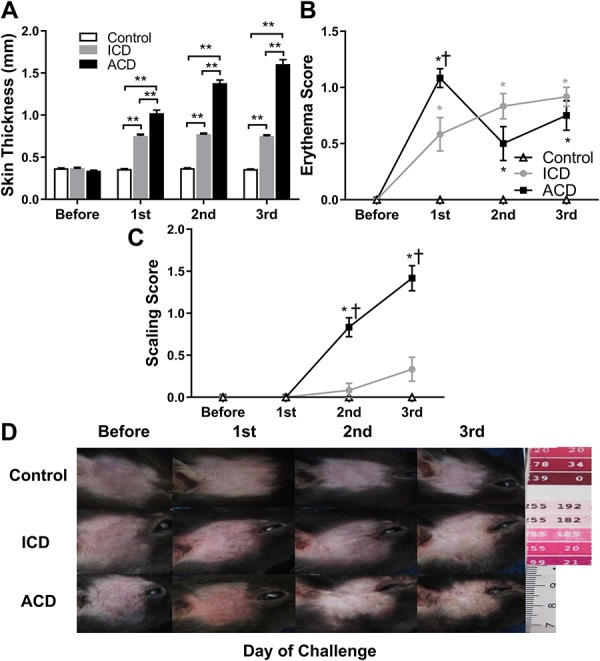
Effects of ACD and ICD on skin thickness and visible signs of inflammation of skin at the challenge site on the cheek. The mean thickness of a fold of cheek skin measured with a micrometer (A) was obtained before and after each challenge with SADBE (ACD or ICD) or acetone vehicle (control). Clinical assessments of erythema (B) and scaling (C) were each separately scored on a scale from 0 (none) to 4 (very marked) (see Methods). In (A), *P < 0.05, **P < 0.01 indicating significant differences between groups, and in (B and C), *P < 0.05 for significance compared with the control group, †*P* < 0.05 indicating significant differences between ICD and ACD groups, error bars: SEM. n = 12 male mice/group. (D) Exemplary photographs that best represent the visible change of erythema and scaling of the skin of the cheek. Photographs were obtained from different mice tested under a particular experimental condition and point in time. ACD, allergic contact dermatitis; ICD, irritant contact dermatitis.

Control mice exhibited no change in skin thickness after each challenge with acetone. The skin thickness increased significantly after the first SADBE challenge for both ACD and ICD but more so for the former. The skin thickness remained the same for ICD mice but increased significantly for the ACD mice after each subsequent treatment (Fig. 4A). Allergic contact dermatitis mice had significantly thicker cheek skin than ICD mice, after each of the 3 challenges (Fig. 4A). Similar differences in skin thickness after SADBE challenge were obtained from the calf (supplemental Fig. 2A, available as supplemental digital content at http://links.lww.com/PR9/A50).

For control mice, there were no visual indications of redness (Fig. 4B), scaling (Fig. 4C), or swelling before and after each application of the acetone vehicle (Fig. 4D). By contrast, challenge with SADBE produced a noticeable swelling of the skin for both ACD and ICD mice. Irritant contact dermatitis mice displayed some redness after the first challenge and a slight scabbing in some mice after the third. By contrast, most ACD mice developed an obvious redness after the first challenge with lesions developing after the second and more so after the third (Fig. 4D). There were no differences in the mean scores for erythema, scaling before challenge (Fig. 4B, C). The erythema score showed an increase 24 hours after all 3 challenges in the ICD group while ACD reached a peak after the first challenge (Fig. 4B). The evaluation of scaling showed an increase 24 hours after the second and third challenge both in the ICD and ACD group, and ACD groups had a higher value than the ICD group (Fig. 4C).

### 3.5. Ultrasound images of skin layers and blood flow

**Figure 5.**
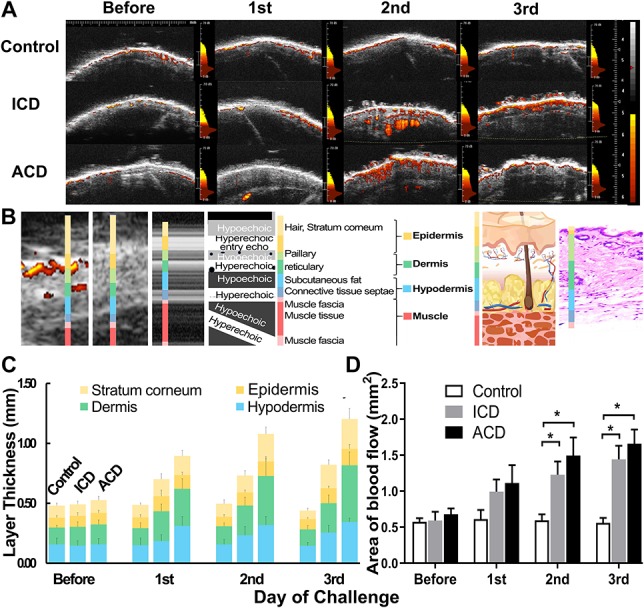
Effects of ACD and ICD on measurements of skin thickness of distinguishable skin layers and blood flow derived from ultrasound images of the cheek. Representative examples of ultrasound skin images of the skin from different mice under different experimental conditions. Colored areas represent moving matter (indicative of blood flow) color coded according to the velocity of movement (35 DB = yellow and 0 DB = red) (A). Ultrasound image from a control mouse illustrating the different layers of skin (B). Mean thickness of each layer of skin (C) and means of areas of blood flow (D) before and each day after challenge in control, ICD and ACD mice. *P < 0.05, error bars: SEM. n = 12 male mice/group. ACD = allergic contact dermatitis; ICD, irritant contact dermatitis.

Applying the method of ultrasound imaging and analysis gave further insight into the daily changes of inflammatory response in these models. Ultrasound images obtained from each group of mice revealed an increase in the thickness of the cheek skin after ICD and ACD but not after acetone alone (Fig. 4A and Supplemental Fig. 2B, available as supplemental digital content at http://links.lww.com/PR9/A50). Similar effects were obtained in measurements of skin-fold thickness of calf skin (Supplemental Fig. 2A, available as supplemental digital content at http://links.lww.com/PR9/A50). There were no differences displayed between different days in the control group and before treatment in the different groups. An increase in skin thickness after the application of SADBE was observed in ICD after the second challenge without further changes after the third (Fig. 5A). The cheek skin was thicker in ACD than in ICD mice after each SADBE challenge, which is consistent with skin thickness measurements with the micrometer (Fig. 4A and Supplemental Fig. 2B, available as supplemental digital content at http://links.lww.com/PR9/A50).The different layers of the skin in the ultrasound images corresponded to those identified from histological tissue sections (Fig. 5B), so that an increase of thickness of all layers was measured for ICD and even more for ACD at the cheek (Fig. 5C and Supplemental Fig. 2C–E, available as supplemental digital content at http://links.lww.com/PR9/A50). In ACD, the most superficial layer increased by 133% in the cheek and the dermal layer by 185% by day 3, whereas the biggest increase in skin thickness in the ICD group was seen in the hypodermis with only 73% increase. Power Doppler analysis revealed a significant increase of dermal blood flow after the second and third challenge in ICD and ACD (Fig. 5A, D).

### 3.6. Histological analyses of calf skin

Because major differences in behavior of ICD and ACD mice were apparent after the second challenge on the cheek, this time point was selected for histological measurements. The thickness of the calf skin was measured before and after the second challenge (Supplemental Fig 2A, available as supplemental digital content at http://links.lww.com/PR9/A50), and the results were in accordance with the findings obtained for the cheek. The histological pictures depicted an increase in the percentage of area stained with hematoxylin (violet) compared with the whole tissue sample of the cheek skin after ICD and ACD, with a stronger reaction for ACD (Supplemental Fig. 3A, available as supplemental digital content at http://links.lww.com/PR9/A50). The same development was seen for the infiltration of cells in ICD and ACD, with a higher cell count for ACD vs ICD (Supplemental Fig. 3B, available as supplemental digital content at http://links.lww.com/PR9/A50). A disruption of epidermal structure and an increase of skin thickness as well as edema in different skin layers in ACD was observed (Supplemental Fig. 3A, available as supplemental digital content at http://links.lww.com/PR9/A50).

### 3.7. Protein expression of IL-1β, TNF-α, CXCL10, and CXCR3

**Figure 6.**
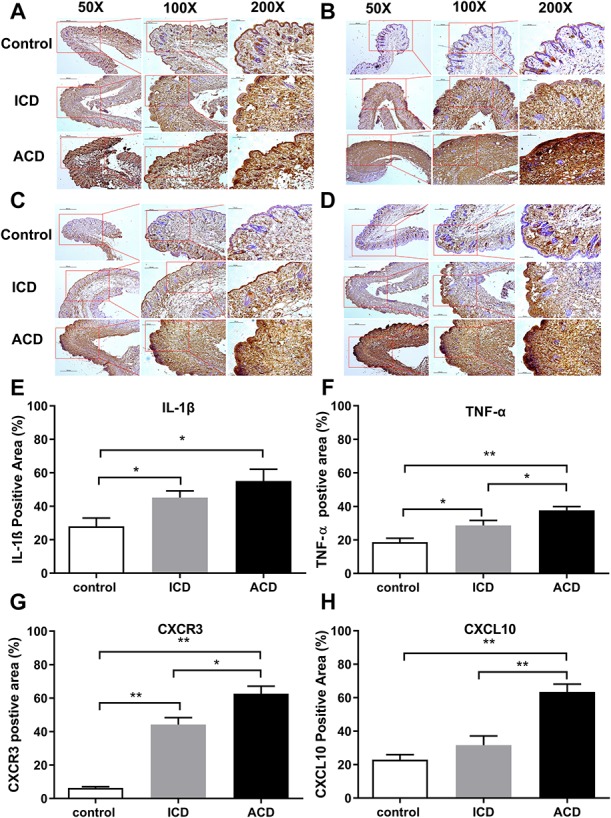
Effects of ACD and ICD on cytokine protein expression in the calf skin. Exemplary images of immunostaining for IL-1β (A), TNF-α(B), CXCR3 (C), and CXCL10 (D) obtained for ACD, ICD, and control mice (shown at magnifications of ×50, ×100, and ×200). ACD, allergic contact dermatitis; ICD, irritant contact dermatitis. Protein expression levels of IL-1β (E), TNF-α (F), CXCR3 (G), and CXCL10 (H) were obtained after the second challenge. *P < 0.05, **P < 0.01, error bars: SEM. n = 3 to 5 male mice/group.

To explore possible biochemical correlates with behavior, immunohistochemical (IHC) staining for protein expression of TNF-α, IL-1β, CXCL10, and CXCR3 in calf skin was obtained on the second day of challenge (Fig. 6A–D). The analysis of protein expression in IHC and the measurements of the percentage of positively stained areas revealed an increase in protein expression in ICD mice in comparison with control for IL-1β, TNF-α, and CXCR3, but not CXCL10 (Fig. 6). By contrast, in ACD, IL-1β, TNF-α, CXCR3, and CXCL10 increased compared with control. ACD had significantly more TNF-α, CXCR3, and CXCL10 in comparison with ICD.

### 3.8. mRNA expression of IL-1β, TNF-α, CXCR3, and CXCL10′

By 24 hours after the second treatment, the mRNA expression of IL-1β, TNF-α, CXCR3, and CXCL10 was significantly greater for ACD than for ICD and greater than control (Supplemental Fig. 4, available as supplemental digital content at http://links.lww.com/PR9/A50). Irritant contact dermatitis levels were not significantly changed from those of control.

## 4. Discussion

In this study, both itch- and pain-like spontaneous behaviors were greater for ACD than for ICD. These results contrast with those in which SADBE-induced ICD and ACD on the ear of the mouse produced the same degree of spontaneous itch-like behavior.^10^ In that study, spontaneous pain-like behaviors were not presented but said to be absent for either ICD or ACD. Methodological differences may have contributed to the different results in the 2 studies. First, we used a higher concentration of SADBE (1% vs 0.5). Second, we used the cheek model and included, in addition to itch-like scratching with the hind limb, the measurement of pain-like wiping with the forelimb using 4 mirrors to obtain all views without which wiping can often go undetected. Third, we tested a larger sample of mice including both sexes.

The present association of pain- with itch-like behaviors of ACD in mice is consistent with a previous finding that SADBE-induced ACD in humans was accompanied by nociceptive sensations of pricking/stinging and burning.^31^ In addition, patients with ICD report experiencing pain and sometimes itch sensations, while ACD patients typically report both itch and pain.^4,6,17,31^ We also presently found that itch- and pain-like spontaneous behavior gradually increased after each challenge in ACD, but not ICD mice. These results indicate that the degree of itch and pain might provide 1 way of distinguishing between ACD and ICD in mice and potentially in diagnostic tests of these disorders in humans.

In addition, behavioral signs of allodynia and hyperalgesia to punctate mechanical stimuli and to heat were stronger for ACD than ICD mice. Human subjects also became hyperalgesic to punctate mechanical stimuli and to noxious heat after ACD induced by 1% SADBE.^31^ Similarly, patients with chronic itch diseases, such as atopic dermatitis, can have additional pain sensations and exhibit hyperalgesia to punctate mechanical stimuli.^1,47^ Whether allodynia and hyperalgesia are absent for SADBE ICD in humans as in the mouse remains to be tested. It seems likely that stimulus hypersensitivity would develop if the irritant inflammation is strong enough. Humans can develop hyperalgesia to mechanical and heat stimuli in response to ICD produced by a sufficiently high dose of the irritant, sodium lauryl sulphate.^32^

Allergic contact dermatitis not only elicited more itch- and pain-like behaviors than ICD in mice but also a greater upregulation in the expression of mRNA and protein for IL-1β, TNF-α, CXCL10, and CXCR3. IL-1β and TNF-α are proinflammatory mediators that are essential for the development of hapten-induced ICD and ACD.^22^ It was previously found that the severity of hapten-induced ICD was related to the level of IL-1β mRNA expression after contact with DNCB and that ACD did not develop in the absence of IL-1β.^2,7,33^ TNF-α upregulation is also essential as the injection of anti–TNF-α antibodies prevented hapten-induced ICD and ACD.^33^ CXCL10, a ligand for CXCR3, is produced by keratinocytes after stimulation with primary cytokines such as TNF-α.^16^ Our finding that this chemokine was upregulated in the skin during ACD but not ICD is consistent with previous observations for mice^11,27^ and for humans.^18^ The upregulation of CXCR3, especially in ACD, likely reflects the infiltration in the skin of the hapten-specific T cells and certain innate immune cells known to express this receptor.^5,41^

In addition, pruriceptive dorsal root ganglion neurons innervating an area of SADBE ACD in mice upregulate CXCR3 and become responsive to CXCL10.^35^ A CXCR3 antagonist blocked the spontaneous site-directed scratching evoked by CXCL10 but not wiping.^35^ It was hypothesized that during the development of ACD, a cytokine from the skin such as TNF-α or IL-1β may have been retrogradely transported to the dorsal root ganglion, resulting in an upregulation of chemokine signaling and an increased density of voltage-gated sodium currents. These effects, in turn, may have contributed to the enhanced spontaneous and stimulus-evoked itch and pain behaviors in ACD mice.^34^ That some or none of these effects might occur with SADBE-induced ICD could be predicted from the lesser upregulation of cytokines and absence of CXCL10 in skin. The effects of anti–TNF-α antibodies or a CXCR3 antagonist on the behavioral effects of ICD and ACD would be important future directions to pursue.

Additional investigations in the future will be required to determine the effects of sex and multiple time points in determining postinflammatory differences in mRNA and protein levels between ICD and ACD. Our postinflammatory analyses were obtained for male mice and only after the second challenge.

Ultrasound imaging and blood flow measurements have been used both in the clinic and in basic research^19,23,30^ and used to noninvasively monitor blood flow, changes in skin thickness, and pathological changes of the skin such as the formation of lesions.^14,50^ Our findings of increased skin thickness, and dermal blood flow point to increased vascular permeability and vasodilatation in the inflamed skin of mice with ICD or ACD. Assessments of the severity of cutaneous inflammation obtained by clinical scoring, ultrasound, histology, and measurement of skin thickness revealed the presence of edema, swelling, disruption of epidermal structures, and strong infiltration of immune cells in ACD. The increased skin thickness and the infiltration of lymphocytes especially in the dermis are manifested as an increase in thickness of the dermal layer.^23^ As expected, these results were more severe in the ACD group than in the ICD group, but both reflect the inflammatory responses in the skin.^45^

In conclusion, SADBE-induced ACD can be differentiated from ICD by greater increases in site-directed itch- and pain-like spontaneous behaviors and also by the presence of allodynia and hyperalgesia. We further showed that these behavioral differences are associated with greater changes for ACD in skin thickness, skin perfusion, upregulation of mRNA, and the levels of protein expression of TNF-α, IL-1β, CXCR3, and CXCL10. The behavioral differences between the preclinical models of hapten-induced ICD and ACD may provide the basis for new approaches to translational research, diagnostics, and therapy for itch- and pain-associated diseases.

## Disclosures

The authors have no conflict of interest to declare.

This work was supported by NIH NS014624 (R. H. LaMotte) and CNNSF 81373993 (H. Nie). N.M. Malewicz received a Research Fellowship (326726541) by the German Research Foundation (DFG). Z. Zhang is the recipient of a scholarship from Jinan University, China.

Part of the data in this article was presented at IASP in Boston in 2018.

## Appendix A. Supplemental digital content

Supplemental digital content associated with this article can be found online at http://links.lww.com/PR9/A50.
